# T Cell Interstitial Migration: Motility Cues from the Inflamed Tissue for Micro- and Macro-Positioning

**DOI:** 10.3389/fimmu.2016.00428

**Published:** 2016-10-14

**Authors:** Alison Gaylo, Dillon C. Schrock, Ninoshka R. J. Fernandes, Deborah J. Fowell

**Affiliations:** ^1^Department of Microbiology and Immunology, David H. Smith Center for Vaccine Biology and Immunology, Aab Institute of Biomedical Sciences, University of Rochester, Rochester, NY, USA

**Keywords:** T cell, motility, migration, inflammation, chemokines, extracellular matrix proteins, CNS, multiphoton imaging

## Abstract

Effector T cells exit the inflamed vasculature into an environment shaped by tissue-specific structural configurations and inflammation-imposed extrinsic modifications. Once within interstitial spaces of non-lymphoid tissues, T cells migrate in an apparent random, non-directional, fashion. Efficient T cell scanning of the tissue environment is essential for successful location of infected target cells or encounter with antigen-presenting cells that activate the T cell’s antimicrobial effector functions. The mechanisms of interstitial T cell motility and the environmental cues that may promote or hinder efficient tissue scanning are poorly understood. The extracellular matrix (ECM) appears to play an important scaffolding role in guidance of T cell migration and likely provides a platform for the display of chemotactic factors that may help to direct the positioning of T cells. Here, we discuss how intravital imaging has provided insight into the motility patterns and cellular machinery that facilitates T cell interstitial migration and the critical environmental factors that may optimize the efficiency of effector T cell scanning of the inflamed tissue. Specifically, we highlight the local micro-positioning cues T cells encounter as they migrate within inflamed tissues, from surrounding ECM and signaling molecules, as well as a requirement for appropriate long-range macro-positioning within distinct tissue compartments or at discrete foci of infection or tissue damage. The central nervous system (CNS) responds to injury and infection by extensively remodeling the ECM and with the *de novo* generation of a fibroblastic reticular network that likely influences T cell motility. We examine how inflammation-induced changes to the CNS landscape may regulate T cell tissue exploration and modulate function.

## Introduction

**Figure 1:**
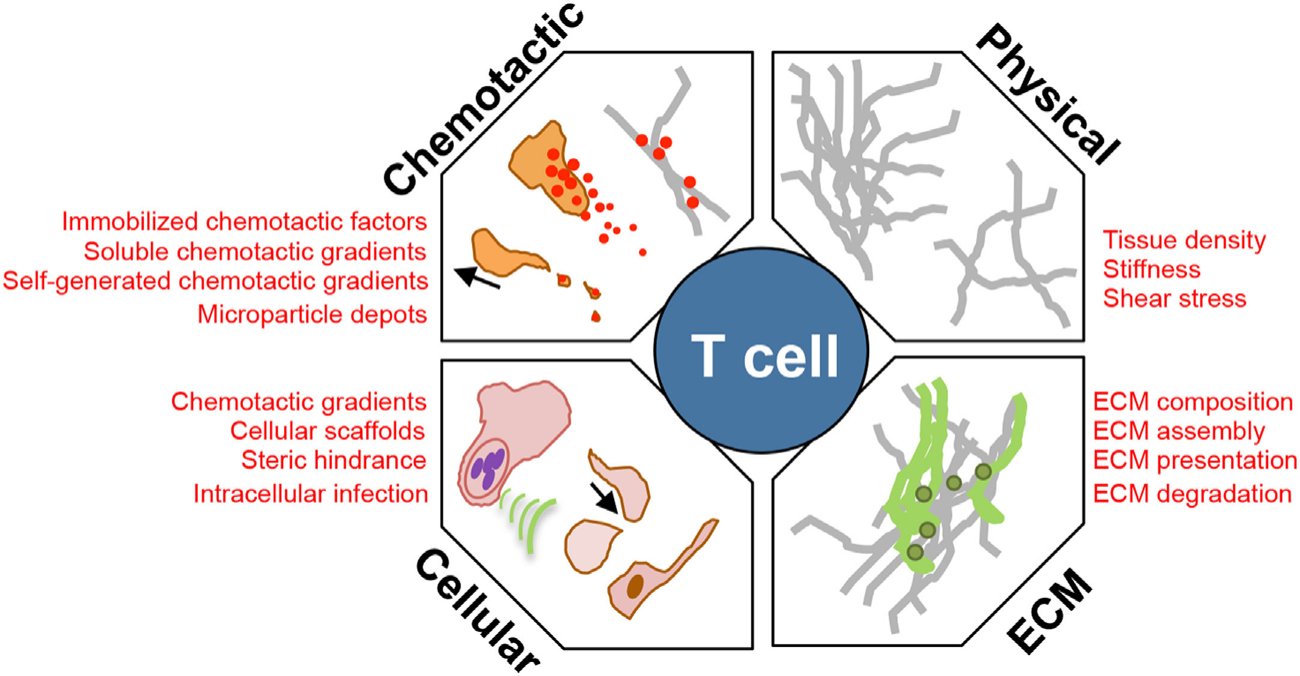
**Environmental modulators of T cell interstitial migration**. T cells enter inflamed sites and must scan the interstitial tissue to locate areas of tissue damage or infection. Their efficiency of interstitial migration is influenced by: (1) the physical structure, level of confinement, and stiffness of ECM; (2) composition of the ECM, collagen fiber-associated matrix proteins, such as fibronectin; (3) cellular composition of the tissue providing: a cellular surface for traction, a source of chemotactic signal, steric hindrance, and a cellular host for pathogens that manipulate the immediate microenvironment; and (4) chemokinetic or chemotactic factors, associated with the ECM, or as a soluble gradient, or within cellular membrane fragments.

The immune system’s success relies on its ability to survey and rapidly respond to infection or damage throughout the body. This task depends on the efficient movement of leukocytes within and between diverse tissues. In recent years, the ability to visualize this dynamic migration using intravital imaging has led to new insights into the cellular interactions between leukocytes and the tissue stroma, T cell “search” patterns within inflamed tissues and the molecular mechanisms that control leukocyte motility and positioning (1). Innate and adaptive immune cells have distinct functional roles as part of a coordinated immune response and must move within complex tissues that are often extensively remodeled by inflammation. Therefore, it is not surprising that mechanisms of motility differ between immune cell types and differ for a given cell type depending on the context-dependent array of environmental cues it encounters. Here, we focus on T cell interstitial motility but take our “cues” from elegant studies on dendritic cell (DC) and neutrophil motility dynamics. How leukocytes integrate and interpret the cacophony of signals coming from their tissue locale into “go” signals during migration and “stop” signals for cell–cell interactions is yet to be fully understood (Figure 1). T cells must traverse their immediate tissue terrain (micro-positioning) as well as accumulate at specific focal sites of infection or damage within inflamed tissues (macro-positioning). Determining whether the cues for these related actions are shared or distinct will be critical to fully understand *in situ* T cell function.

The T cell response is initiated in lymph nodes (LNs) that drain sites of infection or inflammation. T cells are activated by antigen-presenting cells (APCs), mainly DCs, that have migrated from the infected tissue carrying pathogen-derived products presented as peptides in the context cell surface MHC molecules. The APCs also convey information on the type of pathogen or inflammation that they have encountered in peripheral tissues. Through the secretion of specific cytokines, DCs drive the differentiation of T cells into functionally distinct effector cells (Th1, Th2, and Th17) that are better equipped to clear specific pathogens (2, 3). Effector T cells also exit the LN better prepared to interact with the inflamed vasculature through upregulation of adhesion molecules and chemokine receptors (3). As reviewed elsewhere (4, 5), there is now a well-defined series of steps for leukocyte extravasation, the spatiotemporal kinetics of which have been greatly aided by dynamic intravital confocal and multiphoton microscopy. Once T cells cross the vascular and basement membrane barriers, they are met with an often chaotically organized inflamed interstitium. Effector T cells must scan and localize to the area of infection or damage to exert their effector function. Although LN-instructed tissue-specific homing cues provide some preprogramed localization bias (6–9), the inflamed endothelium appears to promote the non-selective entry of a host of different effector T cells. These effector T cells enter an inflammatory landscape unlike any tissue structure they have previously encountered and must utilize cell-intrinsic motility machinery and environment-specific cues to “explore” the new space. We know little about this process for T cells, but studies on innate immune cell types have revealed remarkably adaptable and coordinated mechanisms that prompt movement within inflamed tissues. DCs have been shown to be extraordinarily adept in their ability to seamlessly adapt to different adhesive substrates for locomotion enabling them to traverse a variety of inflamed microenvironments (10). For neutrophils, interstitial migration is aided by cell–cell communication, in part by neutrophil-release of leukotriene B4 (11) that facilitates collective streaming or swarming of neutrophils to a focal point of tissue damage. How effector T cells navigate through heterogeneous inflamed landscapes is less well-defined, yet, it is a critical final step in pathogen clearance and tissue repair.

## *In Situ* Analysis

The mechanics of leukocyte locomotion have largely been defined using *in vitro* models of 2D and 3D environments, most notably collagen and fibrinogen gels and microchannels. These studies have created basic paradigms for amoeboid versus mesenchymal motility, adhesive versus non-adhesive motility (12, 13), the impact of physical confinement (14), and the response to soluble and immobilized chemokines (15). While useful for defining possible molecular requirements, such engineered 3D matrices fail to reflect the *in vivo* composition of the extracellular matrix (ECM), the combinatorial array of chemokinetic and chemotactic signals or the cellular diversity in a given tissue. Importantly, the *in vitro* models do not address the impact of inflammation on such tissue complexity. Indeed, this fundamental difference was highlighted in our recent intravital multiphoton studies of Th1 interstitial motility in the microbially inflamed dermis (16). It is widely thought that leukocyte interstitial motility in 3D environments is not dependent on integrin-based adhesive locomotion (12, 17–19). This notion has been supported by a number of comprehensive *in vivo* studies demonstrating that motility of DCs and neutrophils in the skin (steady state or acute injury), and T cells in the LN, can indeed be integrin-independent events (11, 20, 21). In contrast, we found that Th1 cell motility closely followed the ECM fibers in the inflamed dermis and was dependent on T cell expression of the matrix-binding integrin α_V_ (in combination with β_1_ and/or β_3_). The discrepancy between *in vitro* and *in vivo* studies over the need for integrin-based motility likely reflects the impact of inflammation on tissue remodeling. Adjuvant-induced inflammation in the skin led to a change in the density of the collagen fibers in the dermis, in the deposition of the ECM components, and in the recruitment of innate cell types. Thus, the changes in the tissue landscape *in vivo* during inflammation are complex and multifactorial, and quite distinct from the simplified artificial matrices used in *in vitro* studies. Identifying distinct environmental components, which dictate a dependency on specific molecular machinery for motility in the context of such complexity will be important. With the growing number of intravital studies in inflamed and infected tissues, it is likely that we will continue to see challenges to the current concepts of 3D interstitial migration.

**Figure 2:**
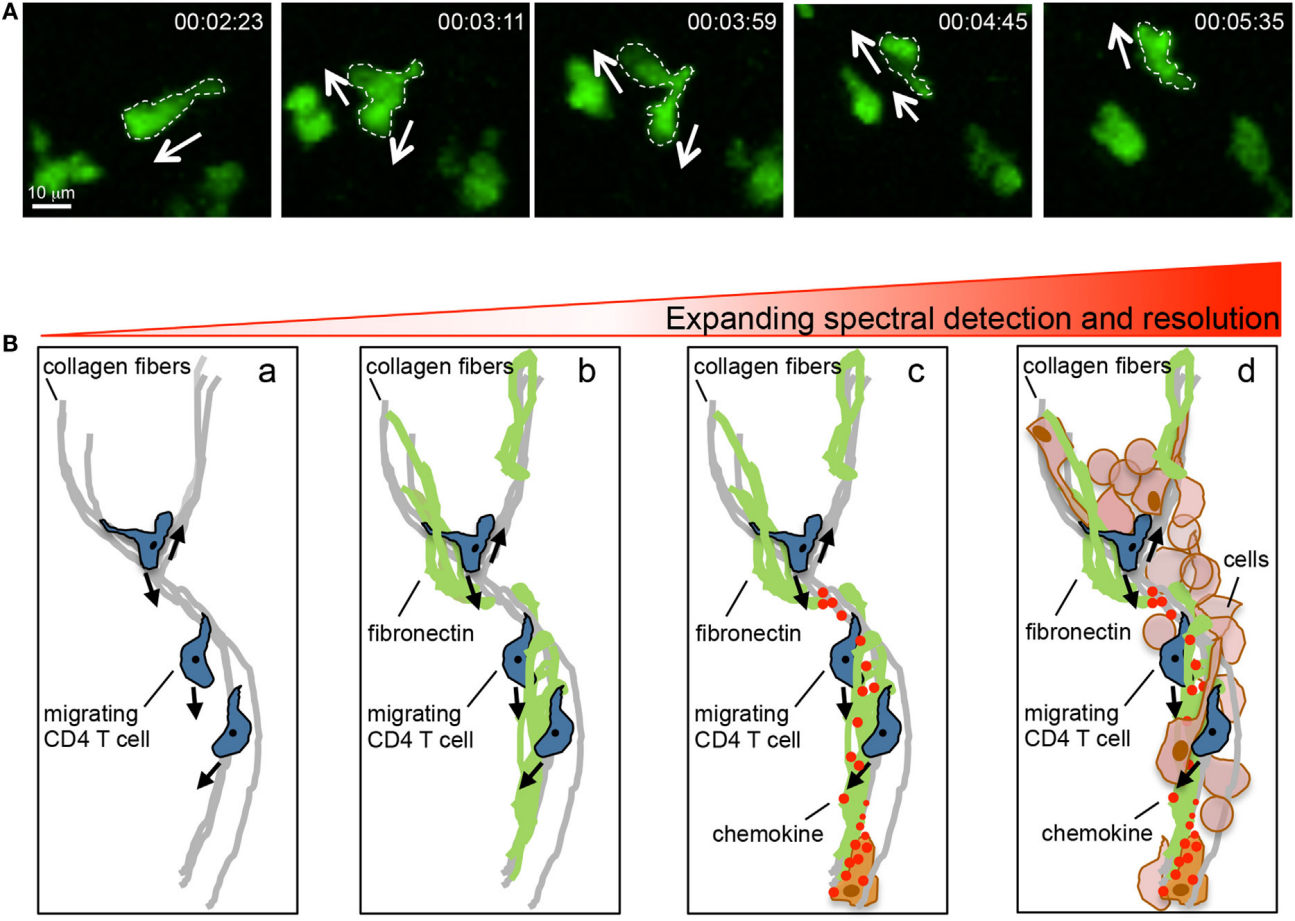
**Intravital multiphoton imaging and its limitations**. **(A)** Motility patterns of effector CD4 T cells in the inflamed dermis showing micro-positional shape changes consistent with dynamic information sampling. **(B)** Imaging capabilities and limitations. Current intravital multiphoton analysis utilizes the SHG signal to highlight the fibrillar core of the tissue matrix and the association of migrating T cells with this structure (a). However, the fibrillar core is cloaked in numerous ECM proteins that cannot, at present, be visualized in real time (b). Moreover, the directional decision making is influenced by chemical signals presented on the ECM, these factors are also optically silent in current real time imaging (c). An additional layer of complexity is provided by the host of stromal and immune cells that are present in the inflamed tissue (d). Our current multiphoton capacity may allow for the detection of the SHG signal in combination with analysis of the interaction between two (may be three) additional fluorescently tagged cell types. But the ability to visualize the quality of these interactions through probes that illuminate signaling events is limited both in optically separable colors and in resolution.

Intravital microscopy has proven a powerful tool for the dissection of spatiotemporal behaviors of leukocytes *in situ*. Numerous observations in the past 15 years have led to novel insights into immune function that had not been predicted from conventional static measures (Table 1). Yet, there are limitations to our current intravital investigative abilities *in vivo*. Multiphoton (two and three photon) microscopy has provided the depth resolution to begin to examine tissues *in situ*. However, these studies are only as good as the structures or cells that can be illuminated with fluorescent probes or with optical effects such as second-harmonic generation (SHG) or use of endogenous tissue fluorescence [elastin, keratin, FAD, and NAD(P)H] (22, 23). Multiphoton constraints come from single or dual laser systems that limit the number of fluorophores that can be simultaneously excited, thus, restricting the complexity of structures and cells that can be visualized in a given field at the same time. In addition, with respect to the ECM, there is limited capacity to label these moieties in real time. Current approaches heavily rely on intravital multiphoton detection of fibrillar collagen with SHG, but this is likely only to reveal a skeleton of the ECM. Fixed tissue immune-histochemical techniques have revealed that these collagen structures are often enveloped by other matrix components, such as fibronectin and lipid moieties, which are optically silent in current multiphoton studies (16, 24, 25). In the brain, defining the ECM structure in real time is particularly challenging as the ECM structure is often non-fibrillar and hence not visualized by SHG. Thus, dynamic imaging of leukocytes is only as good as the ability to define the optically dark “black” space surrounding the cells of interest (Figure 2). The actual matrix and/or cellular structures over or between which T cells move in the inflamed interstitium remain poorly defined. The ultimate goal will be to generate a topographical map of the inflamed tissue to assess the structural, chemical, and cellular contributions that act to guide interstitial T cell scanning and positioning. Moreover, while we can intensely interrogate the micro-positioning cues measured over short distances (200–500 μm) for short periods of time 2–3 h, intravital dissection of the macro-positioning (400–800 mm) that likely takes place over a much longer timeframe (8–12 h) is challenging. Additionally, while multiphoton imaging allows for visualization of structures and cells deeper within tissues than confocal or epifluorescent imaging modalities, the resolution of multiphoton imaging is limited by long excitation wavelengths and asymmetric distortion of laser pulses, compromising *intravital* motility analyses such as the intracellular redistribution of molecules during migration and cell–cell interactions (26). Recent advances that combine photoactivation or photoconversion systems with multiphoton imaging will allow for pinpoint fluorescent labeling of a given cell or groups of cells in a given location in a tissue for assessment of long-range spatiotemporal dynamics, as recently shown for lymphocyte exchange between B cell follicles in the LN (27).

**Table 1:** **Novel insights into leukocyte function from intravital imaging**.

Discovery	Reference
Naïve T cell:APC dynamics. First look at the initiating events in T cell activation showing distinct phases of short and longer interactions	(127)
Stromal cell networks guiding T cell LN migration. Evidence that T cells utilized the fibroblastic reticular network for movement within the LN	(34)
T:B cell dynamics at the T/B border. Motile T:B conjugates led by the B cells and controlled by T cell SAP	(128, 129)
Lévy walks for CD8 T cells. Dynamic imaging revealed that T cells migrate in a random walk pattern that may enhance search capacity for rare targets	(45)
Neutrophil swarming. Evidence that neutrophils communicate *via* an intercellular relay mechanism for long-range directional guidance	(11)
Lymphocyte trafficking between B cell follicles. Demonstrated that T_FH_ cells move between multiple germinal centers, potentially enhancing the antibody repertoire	(27)
Intravascular leukocyte function. Neutrophils and CD8 T cells can perform antimicrobial functions without leaving the vasculature	(89, 130)
Chemokines in T:APC interactions. CD8 T cells required expression of CXCR3 to efficiently contact and kill virally infected cells in the skin	(69)
Neutrophil trails. Neutrophils deposit chemokine-rich membrane fragments that enhance CD8 cell accumulation in the influenza-infected lung	(102)
CTL:target cell dynamics. Revealing motile kinases with targets rather than static synapses and a requirement for CTL cooperativity	(131)

## Lessons from Lymphoid Tissues

Before entering inflamed tissues, T cells have undergone a series of activation events in the LN that has armed them with discrete functional properties and the ability to better respond to environmental cues that may be encountered in inflamed tissues. The spatial positioning of T cells within the LN for optimal T cell activation and differentiation has been extensively reviewed elsewhere (3, 28–30) and is not the focus of this current review. However, there are a number of mechanistic concepts that have arisen from the study of interstitial migration of T cells within the LNs that are worth noting here as reference points for our discussion of T cell movement in inflamed non-lymphoid tissues. First, T cells must engage with particular DCs in order to receive activation signals for function. DCs present a variety of peptides in the context of MHC molecules on their cell surface and DCs presenting a given peptide are likely at low frequency, estimated at 1:100 (31). Thus, each antigen-specific T cell needs to scan the cell surface of many DCs before encountering one that is presenting their specific antigen. Whether this is an active “search” or an optimized chance encounter is unclear (32). Second, intravital imaging of the LN has shown that T cell amoeboid-like motility best fits a random walk with no evidence of directional migration over a 400–600 μm span (33). The shaping of such motility patterns to optimize scanning of the LN is likely to be influenced by both T cell intrinsic migratory machinery and extrinsic directional cues. Third, the structural organization of the LN provides a scaffold for T cell migration that optimizes encounters with DCs. The highly organized fibroblastic reticular cell network (34) acts as a cellular platform for chemokine-dependent, integrin-independent, haptokinetic T cell movement and also promotes encounter with DCs by colocalizing T cells with DCs (35). Fourth, T cell effector functions are acquired and refined in spatially distinct locations requiring repositioning within the LN. In recent years, our understanding of the signals for T cell activation and differentiation has been reshaped to incorporate location-specific instructional cues. Differentiation of both Th1 and Th2 cells in the LN is not complete without the relocation of activated T cells from the T cell zone to spatially distinct regions, namely the peri and interfollicular regions (36, 37). APCs in those specific regions provide additional differentiation signals to T cells to complete functional maturation. Chemokine production by the APCs and corresponding chemokine receptor expression by the T cells both appear key to such T cell positioning (28). How these apparent long-range positioning cues relate to the cues for the observed random walk of T cells still needs to be reconciled. The upregulation of specific chemokine receptors may make activated T cells more receptive to APC-derived chemotactic cues, but direct evidence of T cell directional migration in the LN toward the interfollicular region is lacking. If not actively following a chemotactic gradient, it is possible that the chemokine-driven positioning cues may instead act as focal arrest signals for randomly migrating T cells (38). Thus, observations from the LN have highlighted the importance of efficient T cell scanning of tissues, the interface between T cells and the tissue structure, and how tissue location can impact function: all important concepts when considering effector T cell motility in non-lymphoid tissues.

## Micro- and Macro-Positioning in Inflamed Tissues

**Figure 3:**
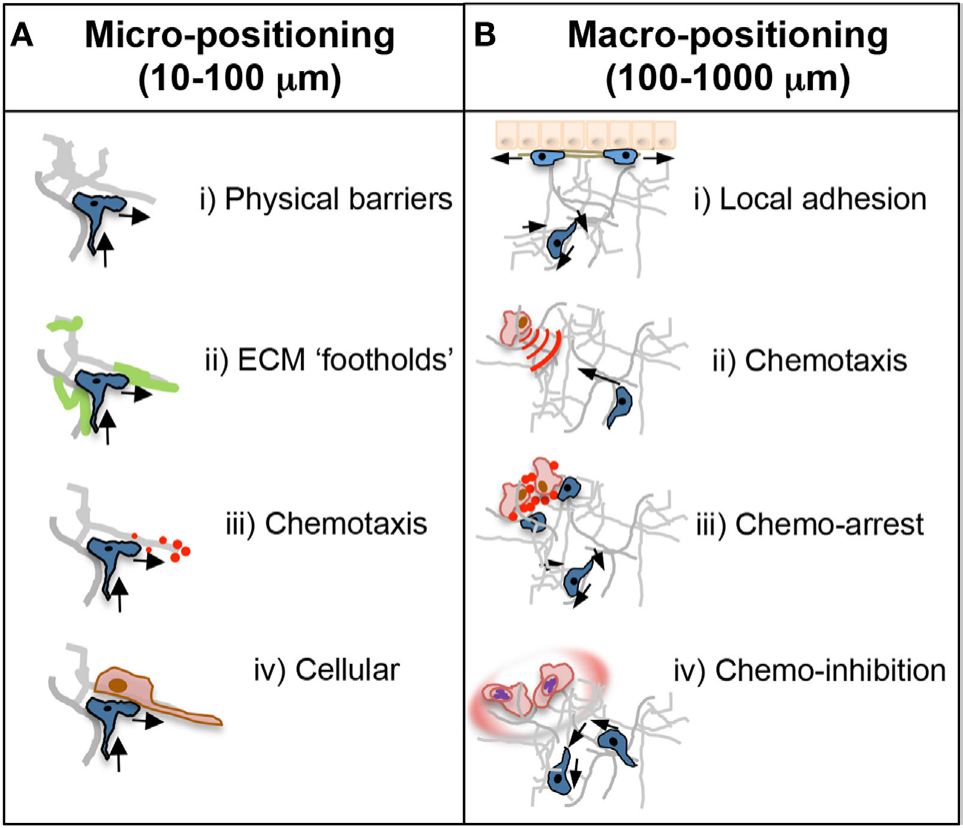
**Micro- and macro-positioning cues**. **(A)** Micro-positioning cues for short-range cell directionality and motility patterns. T cell motility and scanning patterns are influenced by the microanatomical physical and chemical structure of the inflamed tissue. (i) The ECM could provide physical barriers to forward movement in one direction necessitating a change in direction toward a more accessible area. Cells are often seen doubling back on their path at points where the tissue may be impassable. (ii) ECM components may also positively guide directionality as an adhesive substrate that may provide footholds for T cell movement. (iii) Chemotactic cues, such as cytokines, are often presented on the ECM and could dictate scanning patterns and directionality. That T cells often backtrack suggests chemokines may provide a chemokinetic signal in the absence of a directional cue. (iv) The cellular make up of a T cell’s immediate surroundings could influence directionality through steric hindrance or by facilitating migrational paths along cellular projections closely associated with the ECM. **(B)** Macro-positioning cues for long-range movement between distinct regions of a tissue. (i) The mechanistic basis for cellular positioning within a tissue is poorly understood in real time but is controlled in part by distinct expression of adhesion receptors and ligands on individual cell types and at distinct anatomical locations. (ii) Chemokines are also critical regulators of positioning; achieved by active chemotaxis to immobilized, or soluble diffusive gradients, or by self-generated chemotactic gradients. (iii) Alternatively, chemokines can induce cellular arrest or retention to effectively halt T cells in areas of high or uniform chemoattractants such as infection foci. (iv) Not touched on in this review, but important for future study, pathogens often manipulate these chemoattractant signals through decoy receptors or soluble factors that inhibit or disrupt local directional cues for T cell positioning.

The movement of effector T cells within inflamed tissues is critical for their ability to function in the control of infection and in tissue repair. As in the LN, T cells entering the inflamed tissue require encounter with APCs expressing their cognate ligand. The efficiency of APC encounter will depend on a balance between being able to scan a large enough area of the tissue and scanning any given area with sufficient rigor (32, 39, 40). Unlike the LN, most inflamed non-lymphoid tissues do not appear to have an organized fibroblastic reticular cell network that could help to direct T cell scanning along structures that are also sites of APC localization. One might imagine that the infected tissue may require less organizational help to facilitate T:APC encounters because the frequency of both antigen-specific T cells and APCs bearing cognate antigen are enriched at the infection site in comparison to the LN. However, intravital studies in mycobacterial granulomas of the infected liver revealed that antigen presentation was surprisingly limiting (41). Antigen-specific T cells in the infected liver showed few episodes of arrest, often used as a surrogate for T:APC interactions, corresponding to poor T cell activation and limited IFNγ effector function (41). Thus, there is both a conceptual and practical advantage to defining the signals that optimize T:APC encounters in inflamed tissues to enhance effector function and promote pathogen clearance. With respect to pathogen clearance, certain T cell cytokines have a limited range of biological activity, hence interstitial motility must also promote the correct cellular positioning relative to a region of focal infection. In the *Leishmania major* model of cutaneous infection, it was estimated that CD4 T cell production of IFNγ had an 80-micron effective range, measured by the ability of secreted IFNγ to activate nitric oxide pathways in macrophages surrounding the site of T cell activation (42). We suggest that the signals and mechanisms of motility employed for micro-positioning that regulate local T cell maneuvering of the tissue terrain on a micrometer scale may be distinct from the motility that accounts for macro-positioning of T cells at focal points of infection or damage on a millimeter scale (Figure 3).

### Motility Patterns

In lieu of the current ability to visualize the topography of the infected tissues, researchers have initially focused on defining the patterns of T cell interstitial motility and the basic molecular machinery required for locomotion in inflamed tissues (17, 19, 32, 43). In a variety of tissue locations (skin, brain, liver, and gut), and under distinct inflammatory challenges, T cell interstitial motility is amoeboid-like in nature (18) (Figure 2A) and has been likened to a non-directional, random walk (33, 44). Unlike many innate immune cells, there is no requirement for proteolytic cleavage of tissue matrix for motility and no evidence of T:T cell communication for streaming or collective migration. A more detailed analysis of the type of random walk for CD8 T cells in the *Toxoplasma gondii*-infected brain revealed a pattern of random motility that most closely resembled the generalized Lévy walk (45) with a number of small steps for intense exploration of an area interspersed by random longer steps for a wider search area. This pattern is observed across species from sharks to honeybees in the search for rare resources. Indeed, modeling of this T cell behavior revealed that the Lévy walk was more efficient in finding rare APC targets than the random Brownian walk (39, 45). Whether this type of tissue scanning is generalizable to other T cell subsets and to CD8 T cells in different locations is not yet known. However, the observation has sparked much interest in examining the potential link between the T cell “search” for rare APC targets in inflamed sites to models of search theory (32). While the use of “search” evokes a desire or need that probably does not equate to T cell scanning of inflamed tissues for APC, such conceptual parallels are likely to provide interesting hypotheses for future studies.

### Mechanisms of Motility

The movement displayed by effector T cells in lymphoid and non-lymphoid tissues morphologically resembles that of amoeboid migration with cells constantly changing shape with protruding and retracting pseudopods (Figure 2A). Amoeboid migration is driven by the forces generated by polymerization of the actomyosin cytoskeleton and, in its basic form, depends on polymerization of actin for protrusions and type II myosin-dependent actomyosin activity for contraction (17, 18, 46, 47). These forces need traction created by an interacting surface to drive locomotion. Migration on 2D surfaces, such as the vascular endothelium, during extravasation, requires adhesion for traction (48). Integrins are critical for this adhesion step and are dynamically activated at the leading edge *via* inside-out signaling from GCPRs or outside-in signaling in the presence of high substrate availability (49). Regulation of integrin de-adhesion at the trailing edge of the cells *via* myosin II-dependent contraction detaches the cell from its substrate allowing for forward migration (50). In 3D environments, the need for surface adhesion for motility is mitigated by the ability to use opposing surfaces of the tissue matrix for traction in an integrin-independent fashion (14, 51, 52). Leukocytes are thought to use the matrix as a physical scaffold for pushing mediated by actin polymerization at the leading edge and myosin-dependent squeezing at the rear. This non-adhesive locomotion is dependent on the degree of physical confinement afforded by the density of the surrounding tissue matrix (10, 14, 53, 54). As previously mentioned, these lines of distinction between 2D high adhesion and 3D low adhesion modes of motility appear to become blurred in the context of inflammation, with effector CD4+ T cell interstitial motility being dependent on the matrix-binding α_V_ integrins (16). Integrin-dependency for interstitial migration occurred within the context of a tissue matrix that had been modified by inflammation. The inflamed dermis was associated with the fibrillar collagen scaffold (as defined by SHG) becoming less dense than the non-inflamed dermis. One possibility, therefore, is that the change in the matrix density limits the efficiency of a purely biophysical mechanism of force transduction and leads to T cell dependency on integrins for traction. At this stage, it is not clear if the mechanism of T cell interstitial motility has any effect on the efficiency of tissue scanning for cognate antigen. Interestingly, adhesion-based motility has long been thought to be a slower process than non-adhesive amoeboid movement (17, 54). Integrin-based interstitial migration may, therefore, afford the effector T cells, a more thorough scan of the local inflamed microenvironment and may enhance interactions with haptotactic signals.

### Intrinsic Programing for T Cell Interstitial Motility

How micro-positioning decisions are made by T cells as they scan the inflamed tissue (Figure 2A) is unresolved. However, T cells are likely to be guided by their immediate physical and chemical milieu as discussed in the next section. Given, how fundamental the ability to move through inflamed tissues is to T cell function, it has been suggested that T cells may have specific cell-intrinsic mechanisms to optimize interstitial motility. The first demonstration in support of this notion came from the study of a hematopoietic cell-specific myosin, Myosin 1g (Myo1g) (55). T cells express high levels of Myo1g, and it appears to be dynamically relocated at the plasma membrane particularly during migration in 3D environments. In the absence of Myo1g, T cell 3D migration patterns were altered with increased speed and straightness. Using *in silico* modeling alongside *in vivo* imaging, the altered migration pattern in the absence of Myo1g was shown to reduce the capacity of T cells to search for rare APC targets. Thus, through expression of Myo1g, T cells appear to be inherently prepared for efficient scanning of 3D tissues. In addition to a pre-tuned program for T cell motility, T cells may also acquire new interstitial migratory potential during their activation and differentiation in the LN. Studies on the importance of integrin α_V_ for Th1 interstitial motility in the inflamed dermis showed that α_V_ expression is specifically upregulated on those activated T cells destined to exit the LN for effector function in peripheral tissues (16) and also enhanced in Th17 cells in the CNS (56). Therefore, the activation and differentiation process in the LN appears to prepare effector T cells for more efficient interactions with the inflamed matrix for interstitial motility. Distinct cytokine-producing T cell subsets such as Th1, Th2, and Th17 cells most likely need to function in very different inflammatory milieus that are shaped by distinct pathogen challenges. It will be interesting to determine if these cells may be distinctly programed during differentiation to express a unique motility toolbox tailored to efficient interstitial motility in specific inflammatory environments.

T cell-intrinsic programing for macro-positioning within a tissue is evident on numerous levels. In response to herpes simplex virus, CD4 and CD8 effector T cells both efficiently enter the infected dermis but locate to distinct regions of the skin: CD4 T cells to the dermis and CD8 T cells to the epidermis (57). Similar differences in the position of CD4 and CD8 T cells have been seen at other mucosal sites (57–60). Distinct macro-positioning in the skin was not due to differences in interstitial motility patterns between the two T cell subsets but correlated with CD8 T cell expression of the integrin α_E_ (CD103) and epithelial expression of its ligand E-cadherin (57). It is not clear if there is differential T cell directional guidance to the epidermis, or if these interactions simply provide important stop signals for retention in the distinct areas. Similar questions arise when considering the programing differences in chemokine receptor expression between CD4 T effector cell subsets. Functionally distinct Th1, Th2, and Th17 subsets express overlapping, yet, distinct arrays of chemokine receptors that are induced during differentiation (61). The particular chemokine receptors expressed appear to provide unique recruitment and positioning advantages to each subset. Best studied, thus far, is the expression of CXCR3 (ligands, CXCL9, CXCL10, and CXCL11) by Th1 and CD8 cells. CXCR3 is not expressed by Th2 cells and is variably expressed by Th17 cells. CXCR3 has been implicated in CD4 T cell localization to the interfollicular region in the LN to complete Th1 differentiation (36), Th1, and CD8 entry into numerous infected tissues and tumors (62–68) and more recently in the ability of CD8 cytotoxic T cells to locate and kill virally infected cells within the infected skin (69). CXCR3-deficient CD8 T cells were able to enter the vaccinia virus-infected dermis, but fewer CD8 T cells entered, or were retained in, the infection foci compared to WT CD8 T cells (69). Interestingly, of the CD8 T cells that made it into the virus-infected foci, CXCR3-deficient CD8 T cells moved more quickly than CXCR3-expressing CD8 T cells suggesting that CXCR3 facilitates T:APC interactions by decreasing the speed or inducing arrest of the effector T cells. Indeed, elegant studies in zebrafish have revealed that neutrophil motility is restricted in the immediate vicinity of a cellular chemokine source suggesting that, at high or uniform concentrations, chemokines can optimize retention at specific sites in the infected tissue (38). Differential chemokine receptor expression on Th1 (CXCR3+) and Th17 (CCR6+) cells also appears to play a role in autoreactive T cell accumulation in discrete regions of the central nervous system (CNS) resulting in quite different pathological disease (see section below). As will be discussed below, it is unclear if the expression of these chemokine receptors guide micro-positioning during interstitial motility of T cells, or if the macro-positioning of these distinct T cells is guided by focal chemokine gradients or retention signals (Figure 3). Nonetheless, T cell subset-specific chemokine receptor expression appears critical for macro-positioning within the LN and at sites of infection or inflammation. Thus, cell-intrinsic motility programs acquired by effector T cells during activation in the LN equip T cells with the potential to respond and adapt to a variety of environmental cues that may be present in the inflamed target tissue. The utilization of particular motility components within inflamed tissues will be driven by the type of inflammatory-induced changes within the target tissue.

## Environmental Cues

Effector T cells entering an inflamed tissue are met by a cloud of environmental cues from the ECM, lipid moieties, chemokines, cytokines, and purinergic factors, among others. How the T cell integrates and weighs the importance of the different signals for interstitial motility is not clear. On the micro-positioning level, the rapid cell shape changes during effector T cell interstitial motility (Figure 2A) suggest constant information sampling for directional decision-making. There is fairly sparse *in vivo* data on the environmental cues that actually support the motility patterns observed for T cell migration in 3D, hindered in part by the difficulties in visualizing, in real time, the matrix structure and associated chemotactic factors. An emerging theme, however, is that T cells utilize the tissue structure as a scaffold for haptotaxic motility.

### Physical Guidance Cues

The ECM defines the 3D structure of tissues; the organization and composition of which is distinct for individual tissues in the steady state. During inflammation, the ECM is extensively remodeled through the release of cytokines and matrix metalloproteinases, changing the biophysical structure of the matrix, its composition and its “presentation” of bioactive compounds that impact leukocyte motility and function (70–72). The interstitial matrix of many tissues is made up of a core collagen fiber network, the topography of which is shaped by its associating glycoproteins, such as fibronectin, and proteoglycans, such as decorin and versican, that contain glycosaminoglycans (GAGs) subunits. GAGs play important roles in sequestration and display of chemokines and cytokines (73–75). Leukocytes interact with the ECM and can process signals from the physical spacing and composition of the fibers, the rigidity of the matrix (mechanosensing), and immobilized chemical signaling moieties (76, 77). In turn, these signaling events direct leukocyte migration, function, and survival.

Intravital imaging in different tissues has shown that T cell motility closely follows a network of fibrillar structures defined by SHG (16, 78–80). As discussed for actomyosin motility, the density of the ECM is likely to dictate the effective molecular machinery that will facilitate T cell movement. Three-dimensional confinement studies using microchannels revealed a change in the CD8 T cell migration efficiency based solely on the spacing between fibronectin-coated surfaces, with T cell MyoIIA optimizing T cell motility by limiting surface adhesion (14). *In vivo*, within a given inflamed tissue, T cells are likely to experience a highly heterogeneous physical structure with variable degrees of confinement. Some have suggested that T cells may adapt to these changes by following paths of least resistance (17), while studies from DCs and neutrophils suggest that leukocytes can rapidly adapt to distinct terrains by switching between adhesive and non-adhesive motility (10, 11). The impact of utilizing possible “preferred” paths through a tissue, versus the ability to switch between migration modes, on the efficiency of T cell scanning of a tissue for APC encounter has yet to be determined. To add to the complexity, the actual physical space that T cells navigate within will also be shaped by the cellular composition of the tissue (Figure 2B), thus the true degree of T cell confinement *in vivo* is difficult to predict. In the LN, T cells can migrate along the cellular FRC network, where fibroblasts envelop a collagen core. In non-lymphoid tissues, both fibroblasts and macrophages can extend long cellular protrusions that align along the collagen fibers and could also provide a cellular platform for T cell migration. Indeed, macrophage aggregates in liver mycobacterial granulomas appear to provide a cellular scaffold for effector T cell migration (81). The extent to which T cells interact directly with the ECM versus indirectly *via* ECM-associated cells is likely to be context-dependent.

In addition to changes to the structure of the interstitial matrix, inflammation and tissue damage have a dramatic impact on the composition of the ECM, with collagen fibers being decorated with fibronectin, vitronectin, and tenascin. The magnitude and patterning of ECM deposition likely plays a role in both T cell micro- and macro-positioning (Figure 3). In contexts where matrix-binding integrins facilitate T cell interstitial motility, the microanatomical display of the matrix ligands may guide T cell motility patterns by providing local “footholds” or may vary the efficiency of scanning by impacting the speed of T cell movement due to variation in traction. Effector T cells express a variety of matrix-binding integrins that provide ligand specificity for distinct matrix components (49, 82–84). The relative expression of particular matrix-binding integrins differs between inflamed tissues. In the skin, CD4 effector T cells predominantly express α_2_β_1_ and α_V_β_1_/β_3_, while in the lung and gut effector, T cells express a far wider variety of matrix-binding integrins (16). For skin and lung, the differences in integrin expression appear to correlate with the complexity of the matrix landscape. Inflammation in the skin led to a broad distribution of fibronectin across the dermis, while in the influenza-infected lung, there were spatially distinct regions that where either fibronectin-rich or collagen-rich (16). These compositionally distinct ECM regions within a tissue may afford distinct macro-positioning cues for local function or retention (3, 59, 85–88).

Our discussion has focused on the ECM as a physical facilitator of T cell interstitial migration. However, studies coming from the tumor field highlight the potential barrier function of a remodeled ECM. Real-time imaging of human lung tumor slices revealed that the density and orientation of the ECM fibers surrounding the tumor mass directed T cell migration around the tumor but restricted them from entering the tumor mass itself (80). Recent intravital imaging of CD8 T cells in the liver demonstrated the novel ability of CD8 T cells to sample the subsinusoidal hepatocytes and kill virus-infected hepatocytes, without exiting the vasculature (89), a function that was inhibited by changes to the liver structure during fibrosis. The degree to which the ECM imposes a physical restriction on T cell access during chronic infection and inflammation is unclear and warrants further investigation.

### Chemical Guidance Cues

Leukocytes can respond to multiple chemoattractants within the inflamed tissue including chemokines, cytokines, lipids, ECM fragments, and puronergic signals. For T cells, much of our understanding of chemotactic signals have come from the study of chemokines, chemokine-receptor expression, and the blockade of receptor signaling using GPCR inhibitors such as the G_i_ inhibitor, pertussis toxin. Interference at each of these levels has reinforced that chemokines are major positioning cues for T cells in the steady state and during infection and inflammation (35, 61, 90, 91). While it has been assumed that diffusive chemokine gradients provide chemotactic cues for T cell directed migration, direct evidence for chemokine gradients on a micro- or macro-scale is limited (38, 92, 93). A subtle directional bias was observed for migration of CD8 T cells toward HSV-1-infected cells in the skin, but cells moved away from the infection site almost as often as moving toward infected cells (94). The weak directional cues and the often observed patterns of random T cell motility using intravital imaging raise the possibility that chemokines direct T cell positioning in ways other than through classic concentration gradients (44).

For many chemokines, their activity is dependent on correct presentation by GAGs associated with the ECM or cell surfaces. While intravital studies utilizing pertussis toxin treatment have shown a dependency on G_i_-linked signaling for interstitial migration, how the chemokines support T cell motility is unclear. Blockade of the CXCR3 ligand CXCL10 in the *Toxoplasma*-infected brain reduced the velocity of CD8 T cells but did not disrupt the Lévy walk pattern of movement in the tissue (45). Thus, chemokines may not shape the pattern of T cell motility but, rather, optimize the speed of interstitial migration, which may in turn increase the rate of T:APC chance encounter. This could be achieved through a basic chemokinetic mechanism or through activation of matrix-binding integrins, akin to the well-established role for chemokines in integrin activation and ligand binding on the vascular endothelium (95). More recently, an alternative mechanism has been proposed, that of self-generated chemotactic gradients (96). The model proposes that cells can form their own chemical gradient by degrading a local source of attractant. *In vivo* evidence for such a mechanism first came from studies of the migrating primordium of zebrafish where, in the presence of uniform expression of SDF-1, a signaling gradient across the primordium was achieved by sequestration of SDF-1 at the rear by the receptor CXCR7 (97, 98). Recent examples in the LN and spleen suggest that decoy receptors or metabolizing enzymes expressed by immune cells themselves remove or degrade the attractants to create local gradients for lymphoid migration (99–101). This potential mechanism may have distinct advantages for an effector T cell’s “search” of an infected/inflamed tissue as it can be effective over a wide range of attractant concentrations enabling long-range self-directed exploration. Moreover, by regulating the expression of distinct scavenging receptors, particular effector T cells may separate the functionally important signals from the multitude of chemotactic signals in an inflamed tissue.

A recent study of neutrophils in influenza-infected mice reveals an additional layer of T cell migratory control (102). Neutrophils crawling in the interstitium of the infected trachea left long-lasting membrane fragments behind that were enriched for the chemokine CXCL12. Such chemokine depots deposited by the neutrophils appeared to provide guidance cues for incoming CD8 effector T cells for motility and effector function. These novel findings suggest that T cell interstitial migration may be shaped by the preceding recruitment and interstitial migration of innate cells that leave chemotactic trails for subsequent T cell movement.

The lack of evidence for macroscale directional migration leaves open the question of how chemokines guide cells to specific locations within a complex tissue. Common to many studies on T cell positioning is the presence of a location-specific cellular source of critical chemokines. Stromal cells in the interfollicular region of the LN were potent sources of CXCL9 required for Th1 intranodal repositioning during Th differentiation (36). Similarly, CXCL9 and CXCL10 were enriched in the vaccinia virus-infected cells in the skin and enhanced CD8 T cell positioning (69). As discussed in the context of CXCR3 expression, these sources of high chemokine production may, instead of being chemotactic, provide signals for T cell arrest and/or retention (38). Indeed, the loss of CXCR3 expression on CD8 T cells led to accelerated movement within the infection foci (69). Mechanistically, calcium signaling, possibly downstream of chemokine receptors, has been implicated in T cell arrest (103). High local concentrations of chemokines may additionally enhance inside-out activation of matrix- or cell-binding integrins, mediating strong adhesion and arrest to cellular or ECM structures (95). Furthermore, chemokines, possibly *via* receptor mediated tethering, have also been shown to promote T cell activation upon APC encounter (104, 105); in effect doubling down on the “stop” signal by optimizing T cell signaling with cognate APCs.

In conclusion, the mechanisms that facilitate efficient T cell interstitial motility in inflamed tissues are, in part, shaped by initial activation and differentiation events in the LN draining the site of infection or damage. The implementation of specific migratory machinery at the site of inflammation, however, appears highly context-dependent. Efficient T cell scanning to locate infection or damaged foci is guided by the tissue-specific matrix scaffold and optimized, in terms of speed and positioning, by chemotactic and arrest/retention cues.

## Unique Lanscape of the CNS

The CNS is an immunologically unique tissue and thus presents a specific set of challenges and considerations for studying T cell motility and positioning. In the steady state, the composition of the CNS extracellular environment is distinct from most other peripheral tissues, lacking the collagen fiber networks that often impart tissue rigidity and organ-level organization. Instead, the interstitial ECM is composed principally of long hyaluronan chains decorated with proteoglycans and cross-linked by tenascin-R (106). Along with this distinct interstitial ECM, the CNS is also punctuated with perineuronal nets. The perineuronal nets are distinct ECM structures composed of chondroitin sulfate proteoglycans that form dense structures around certain subsets of neurons and provide support and stability to neural connections (107). Together, the CNS ECM provides protection from mechanical stress while supporting the function of the neural network (106). Microglia, CNS-specific cells of the innate immune system, in conjunction with astrocytes, mediate tissue homeostasis and are the first to respond to tissue damage or infection (108). Surveillance of the brain and spinal cord by T cells is rare but critical for control of chronic and latent infection (109). The importance of continual immune surveillance was highlighted following reactivation of latent JC polyomavirus infection and development of a progressive multifocal leukoencephalopathy after blockade of immune extravasation using the anti-α4 integrin antibody Natalizumab (110). Those T cells that are present in the circulating cerebral spinal fluid under homeostatic conditions are enriched for memory T cell markers and CXCR3 expression (111); however, a role for specific receptors in T cell immune surveillance of the CNS has not been defined. The atypical structure of the CNS parenchyma under homeostatic conditions may require distinct mechanisms of T cell interstitial motility from those utilized by T cells recruited to the CNS by inflammation and infection (112).

During inflammation, the CNS interstitial ECM as well as the perineuronal nets undergo substantial remodeling (113). Factors produced by infiltrating immune cells and resident glial cells drive this inflammatory restructuring. Enzymes degrade the HA-rich network, and the production of new ECM components by cells such as astrocytes changes the composition of the CNS ECM, altering its mechanical properties (106, 114). Inflammation-induced upregulation of fibronectin and fibrillar collagens have been shown to potentiate T cell migration in the CNS (115), although it is not known if this is an integrin-dependent process. Changes in the ECM also alter its interactions with other bioactive molecules such as chemokines and cytokines. For example, the proteoglycan decorin is strongly upregulated in CNS injury (116). In other tissues, decorin has been shown to bind TGFβ and inhibit its function (117), while decorin binding to IFNγ or TNFα promotes their signaling capacity (118). Alterations in these particular cytokines could change leukocyte motility by modulating responses to chemokines and altering matrix metalloproteinase activity (119). New *in vivo* observations on the structural changes to the CNS during inflammation have important implications for T cell interstitial exploration and draw parallels to other non-lymphoid and lymphoid tissues. Reports have documented the presence of reticular fiber-like structures that develop in the inflamed CNS. These structures, absent in the steady-state CNS, generate a SHG signal and are observed deep within the cortex, discrete from both the vasculature and the meninges (79, 120). While their molecular and cellular constituency remains unknown, the reticular fibers appear to provide a scaffold for T cell migration within the parenchyma. CD8 T cells were shown to traffic along these reticular fibers in a model of CNS infection with the protozoan parasite *Toxoplasma gondii* (79). Immunohistochemistry revealed a coincident fibrillar distribution of CCL21, suggesting these reticular fibers may represent rich regions of haptotactic guidance through immobilization of chemokines. As with infection in other sites, infection-induced focal chemokine production likely dictates T cell positioning in the CNS. West Nile virus-infected neurons produced CXCL10, which mediated CD8+ T cell parenchymal infiltration, specifically into the cerebellum (121). Intriguingly, CXCR3/CXCL10 deficiency affected parenchymal, but not perivascular T cell numbers, suggesting a differential requirement for this chemokine in recruitment to the CNS versus localization deeper within the tissue.

In non-infectious models of autoimmune inflammation in the brain, location-specific expression of individual chemokines appears to direct preferential CNS recruitment or retention of functionally distinct CD4 effector T cell subsets (122). Th1 effectors mediated inflammation of the parenchyma of the spinal cord, but not the brain, while Th17 effectors supported extensive parenchymal inflammation in the brain, but not the spinal cord (123–125). Not surprisingly, such differences in positioning result in distinct neurological pathologies (122). The mechanisms behind this phenomenon are incompletely understood. Differential entry sites into the CNS likely contribute to location specificity. Expression of CCL20 (a CCR6 ligand) by epithelial cells of the choroid plexus appear to promote CCR6-regulated entry of Th17 cells (126). Whether anatomical differences in the tissue structure or ECM composition at these locations impacts the efficiency of Th1 or Th17 migration is not yet clear. However, a recent study has revealed that Th17 cells in the CNS have elevated expression of the integrin α_V_β_3_ (56). Expression of α_V_β_3_ appears to facilitate integrin:ECM-driven Th17 accumulation and function in the CNS as blockade of α_V_β_3_ binding ameliorated Th17-mediated EAE. Intravital imaging of Th1 and Th17 motility in the CNS will be important to determine whether they differentially utilize the inflammation-induced reticular fibers as guides for haptotactic interstitial migration and/or positional guidance.

## Future Directions

Intravital imaging has only just begun to reveal the dynamic spatiotemporal control of immune function in inflamed tissues. Nonetheless, it has already provided novel insight into T cell interactions with the surrounding tissue and with other leukocytes. Future identification of critical, context-dependent, molecular regulators of cellular migration will play an important role in the development of targeted therapeutics that attenuate leukocyte function in specific immune-mediated disease. The success of these efforts will depend on the ability to understand tissue signals in a combinatorial fashion. This will require visualizing the tissue topography at the level of ECM, immobilized chemical signals, and cellular heterogeneity as well as understanding how immune cells integrate and act on the complexity of such signals emanating from their inflamed surroundings.

## Author Contributions

AG and DS contributed equally. AG, DS, and DF wrote the manuscript. AG, DS, and NF researched the literature, created content, and performed experiments that informed the discussion. DF and NF designed the figures. All authors contributed to the design of the manuscript, discussion of relevant literature, and edited each draft.

## Conflict of Interest Statement

The authors declare that the research was conducted in the absence of any commercial or financial relationships that could be construed as a potential conflict of interest.
